# Protocol for a systematic review and meta-analysis of the impact of cervical cancer prevention initiatives in Ghana

**DOI:** 10.1371/journal.pone.0337011

**Published:** 2025-11-20

**Authors:** Emmanuel Timmy Donkoh, Iddrisu Wahab Abdul, Abraham Kwadzo Ahiakpa, Isaac Williams, Rita Nyaaba Akologo, Stephen Danyo, Chrysantus Kubio, Kofi Effah, Joseph Emmanuel Amuah

**Affiliations:** 1 Screen and Treat Research Group, Centre for Research in Applied Biology, School of Sciences, University of Energy and Natural Resources, Sunyani, Ghana; 2 Department of Mathematics and Statistics, Ghana Communication Technology University, Accra, Ghana; 3 Catholic Hospital, Battor, Battor via Sogakope, Volta Region, Ghana; 4 Volta Regional Health Directorate, Ghana Health Service, Ho, Volta Region, Ghana; 5 School of Epidemiology and Public Health, Faculty of Medicine, University of Ottawa, Ottawa, Ontario, Canada; Addis Ababa University, ETHIOPIA

## Abstract

**Background:**

Cervical cancer, though preventable, remains the second most diagnosed cancer and the primary cause of cancer-related deaths among females in Sub-Saharan Africa. The significance of coordinated screening programmes for reducing the burden of cervical cancer in Africa is not well documented. This systematic review will summarize published reports from key databases, grey literature and programme reports to assess the performance of cervical cancer prevention programmes in Ghana.

**Methods:**

To be eligible for inclusion, interventions must target Ghanaian women with cervical cancer screening and prevention strategies using methods such as visual inspection with acetic acid (VIA), mobile colposcopy, HPV DNA testing, cytology (Pap smear), and treatment approaches such as cryotherapy, thermal ablation, loop electrosurgical excision procedure (LEEP). A comprehensive electronic search strategy will be used to identify studies published since database inception, and indexed in MEDLINE, EMBASE, CINAHL and Web of Science. The search strategy will include MeSH terms (and synonyms) relevant to cervical cancer, screening/treatment methods, geographic focus and implementing institution. We will include searches for grey literature, recognizing the value of programmatic and governmental reports that might not appear in traditional databases. Search results will be summarized in line with PRISMA guidelines. The GRADE (Grading of Recommendations Assessment, Development and Evaluation) approach will be used to evaluate and document evidence certainty for all outcomes, internal validity of included reports, inconsistency, indirectness, imprecision, and publication bias. Where sufficient homogeneity exists among included studies in terms of interventions, study designs, populations, and outcome measures, we will perform a meta-analysis to calculate pooled effect estimates and their corresponding 95% confidence intervals.

**Significance:**

This systematic review will assess the performance and impact of cervical cancer screening and prevention programmes conducted in Ghana to date and identify what contextual strategies have delivered the most impact as well as highlight what gaps remain in our understanding of how a nationwide screening programme can be properly construed for maximum impact.

## Introduction

Cancer of the cervix uteri is the fourth most common cancer and the second leading cause of cancer-related deaths among females [1]. By comparison, it ranks as the topmost cause of cancer-related deaths in Sub-Saharan Africa [2,3]. In the year 2022, approximately 660,000 incident cases and 350,000 mortalities from cervical cancer were recorded globally [4]. The fraction of these estimates attributable to developed countries has shrunk drastically over the years and at least 94% of this disease burden is traceable to developing countries from Latin America, East Asia and sub-Saharan Africa [1].

Despite a decreasing trend in global incidence and mortality over the past few years, cervical cancer continues to be a troubling outcome of persistent high-risk HPV transmission in Sub-Saharan Africa [5,6]. This is largely due to a lack of organized and sustainable national programmes in sharp contrast to countries where well-structured cervical cancer prevention programs, including education, vaccination, and screening with prompt treatment of pre-cancerous lesions exist [7–9].

In Ghana, HPV screening is largely opportunistic with accompanying financial constraints [10–12]. Attempts to eradicate cervical cancer as a public health threat, have been hampered by the failure to improve early disease detection and cost-efficient treatment [12]. While screening is generally considered useful, the significance of *ad hoc* campaigns and screening initiatives is difficult to gauge. Some argue that without appropriate systems linking women who screen positive to care, the net effect of screening may be undesirable. Moreover, important ethical and economic sustainability questions arise in such a context.

An emerging strategy for ensuring the sustainability of screen-and-treat programmes in fragile health systems is to embed these services into existing programmes that are robust, familiar to healthcare staff and are closely related enough to be offered as a comprehensive package [13,14]. However, there is limited evidence on effective implementation strategies for providing cervical cancer prevention and treatment services at scale as part of integrated health services. This leads to the loss of patients to follow-up. Further, the disconnect between sporadic screening campaigns and treatment services worsens the healthcare inequities confronting the most vulnerable who make a livelihood in peri-urban and rural communities as well as high-risk groups such as women living with HIV and female sex workers.

From the year 2000–2003, the Ghana Health Service in partnership with the John Hopkins Program for International Education in Gynaecology and Obstetrics (JHPIEGO), initiated the first Cervical Cancer Prevention (CECAP) programme, under the Safety, Accessibility, Feasibility and Program Effort (SAFE) demonstration project, locally known as the Cervicare Programme [15]. The SAFE/Cervicare project aimed to provide a ‘single visit’ screening with Visual Inspection with Acetic acid (VIA), linked to cryotherapy for suspicious lesions [16]. Another key initiative to address gaps in cervical cancer prevention and training in Ghana came in the form of the establishment of a Cervical Cancer Prevention and Training Centre (CCPTC) in the year 2017 in Battor. The primary aim of the CCPTC was to build human resource capacity across Ghana and in other similar low-resource settings to enable provision of crucial cervical pre-cancer screening and treatment services [14]. Aside these, other reports on screening programmes for cervical cancer prevention in Ghana have been published [17]. Unfortunately, the collective impact of approaches used in these programmes has not been documented [18]. This systematic review, therefore, aims to assess the performance and impact of cervical cancer screening and treatment programmes conducted in Ghana among women from database inception to date to identify what contextual strategies have delivered the most impact and what gaps remain in our understanding of how a nationwide screening programme can be properly construed for maximum impact.

## Methods

### Protocol registration and design

The review protocol has been captured in the PROSPERO database (CRD420251035338). Our systematic review of the available published literature and secondary data on the performance of cervical cancer prevention programmes in Ghana will be guided by the Preferred Reporting Items for Systematic Reviews and Meta-analyses (PRISMA) guidelines, and this review protocol conforms to the PRISMA-P checklist (see Supporting information file 1: S1 Table) [19]. Although future amendments to this protocol are not anticipated, they will be recorded on the PROSPERO database and detailed in the review report as required.

### Literature search strategy and key terms

A structured search will be conducted in line with the Cochrane Handbook for Systematic Reviews of Interventions [20] for all reports on cervical cancer screening and prevention programmes in Ghana (Table 1). We will identify keywords and phrases that accurately represent the various dimensions of the review. Terms such as “cervical cancer,” “cervical cancer screening,” and “”Pap test” or “HPV test” will be selected to address the health condition and screening method, while “screen-and-treat” and “screen and treat” will be chosen to capture studies emphasizing this specific approach. To ensure geographical relevance, the term “Ghana” will be included in search, and key implementing organizations such as “JHPIEGO” and “Cervical Cancer Prevention and Training Centre” (CCPTC) will be added, as these entities are known to have implemented relevant programs in the region. The search will be limited to all reports published since database inception until July 2025. Boolean operators and grouping will be employed to refine the search and ensure precision. By combining keywords with “AND” and “OR” and using quotation marks for exact phrases, we will craft queries that capture studies from various perspectives and sources. The use of MeSH terms and multiple databases will allow us to explore multiple permutations of keywords and expand the reach of our search. Large databases such as PubMed, CINAHL and Google Scholar will be queried. Additionally, relevant programme websites will be consulted to uncover project reports, evaluations, and publications, providing additional insights into programme implementation and results that may not be fully documented in peer-reviewed journals. To ensure comprehensive coverage, we will perform searches for grey literature and policy reports that might not appear in traditional databases. Resources such as WHO reports, Ghana Health Service publications, and ProQuest Dissertations & Theses will be searched for sources of grey literature, offering insights into implementation processes and outcomes often found in non-academic reports. This structured approach will provide a detailed and comprehensive view of the literature on cervical cancer screening and prevention in Ghana.

**Table 1:** Example search strategy (MEDLINE/PubMed).

Search item	Search component	Search Terms
1	Health Condition	“cervical cancer” OR “cervical precancer” OR “pelvic screening”
2	Screening/treatment method	“Pap test” OR “VIA” OR “visual inspection” OR “HPV test” OR “cryotherapy” OR “thermal coagulation”
3	Approach	“Screen-and-treat” OR “screen and treat” OR “screen-and-treat approach” OR “single visit approach”
4	Geographic focus	“Ghana” OR “Africa” OR “SSA”
5	Implementing organization	“JHPIEGO” OR “Ghana Health Service” OR “GHS” OR “Cervical Cancer Prevention and Treatment Centre” OR “CCPTC”

VIA: visual inspection with acetic acid; SSA: sub-Saharan Africa; JHPIEGO: Johns Hopkins Programme for International Education in Gynaecology and Obstetrics.

### Inclusion and exclusion criteria

#### Population.

We will include studies focusing on Ghanaian women screened for cervical cancer or precancerous lesions. Special interest studies on defined populations will be included as long as the participants fall within the target criteria.

#### Intervention.

Included reports must involve the intervention of interest, which shall be cervical cancer screening and prevention campaigns or programmes (e.g., SAFE/Cervicare, CCPTC) using methods such as visual inspection with acetic acid (VIA), mobile colposcopy, HPV DNA testing, cytology (Pap smear), and treatment approaches (e.g., cryotherapy, thermal ablation, LEEP). Reports are not required to include both screening and treatment; however, cervical cancer programmes or interventions that do not involve screening or treatment protocols will not be eligible.

#### Comparator.

Comparisons will be made between different screening programmes and screening/treatment approaches (e.g., VIA vs. HPV DNA testing).

#### Outcomes.

Primary outcomes will include screening coverage, positivity rates (e.g., VIA positivity, hr-HPV prevalence), treatment outcomes, and rates of loss to follow-up. Secondary outcomes will include programme reach, effectiveness, adoption, implementation, and maintenance (assessed using the RE-AIM framework), health worker training outcomes, and barriers to screening/treatment.

#### Types of studies.

We will search for reports on cervical cancer prevention activities (typically related to screening using a standard approach with or without follow-up treatment) in Ghana for inclusion. Published articles reporting on cervical cancer screening and prevention in Ghana from database inception with screening outcomes such as positivity rates, risk factors, challenges, and barriers to treatment and follow-up will be deemed eligible for inclusion. Conference abstracts with details about study population, screening approach, sample size, patient sampling methods and cervical cancer screening and prevention outcomes will also be included for screening. This systematic review will include population-based studies conducted in Ghana or those providing well-defined, disaggregated data analyses of Ghanaian cohorts. Multi-center studies incorporating Ghanaian participants will be eligible for inclusion, provided they report sufficient statistical detail specific to the Ghanaian subgroup. Studies meeting these inclusion criteria will proceed to the initial screening phase, as outlined in the study selection flowchart. Studies failing to meet the inclusion criteria will be excluded, including case reports, narrative or systematic reviews, expert opinions, and studies exclusively reporting data on Ghanaian populations residing outside Ghana. Although review articles will not be included in the primary analysis, a manual search of their reference lists will be conducted to identify additional relevant studies.

### Study selection process

Citations retrieved from the database searches will be imported into Mendeley Desktop (version 2.129.0) for reference management, where duplicate records from multiple sources will be identified and removed using automated and manual deduplication processes. Two independent reviewers will conduct an initial screening of titles and abstracts to exclude studies that are clearly irrelevant or do not meet the predefined eligibility criteria, as outlined in the search strategy. Full-text articles of potentially eligible studies will then be retrieved and independently assessed by two reviewers to confirm adherence to the inclusion criteria, using a pre-designed, standardized checklist to ensure consistency. Studies focusing solely on training programmes will be excluded unless they report data on cervical cancer screening/treatment participation, screening/treatment outcomes, or factors influencing screening/treatment uptake. Any discrepancies between reviewers during title/abstract or full-text screening will be resolved through discussion to reach consensus or, if necessary, adjudication by a third reviewer. Data from included studies will be extracted into a standardized Microsoft Excel template, capturing relevant study characteristics, outcomes, and other predefined variables. In accordance with PRISMA guidelines, the reasons for excluding studies at the full-text screening stage will be systematically documented and reported in a transparent manner, including in the PRISMA flow diagram [19].

### GRADE assessment of the certainty of evidence

The certainty of evidence for each outcome in this systematic review will be assessed using the GRADE approach, which evaluates risk of bias, inconsistency, indirectness, imprecision, and publication bias [20]. Primary outcomes, such as screening coverage, visual inspection with acetic acid (VIA) positivity rate, high-risk human papillomavirus (hr-HPV) prevalence, treatment outcomes, and loss to follow-up rate, will be evaluated for all included studies and programme reports. Secondary outcomes will also be assessed, including programme performance (assessed using the RE-AIM framework for Reach, Effectiveness, Adoption, Implementation, Maintenance) and health worker training coverage. Grey literature, such as programme reports will be evaluated using the AACODS checklist, assessing Authority, Accuracy, Coverage, Objectivity, Date, and Significance [21,22]. Serious methodological limitations, such as non-representative sampling or unclear reporting, will lead to downgrading of evidence [20].

As the majority of studies are expected to be cross-sectional studies, the Joanna Briggs Institute (JBI) Critical Appraisal Tool for Analytical Cross-Sectional Studies will be adopted [23] to evaluate risk of bias, focusing on inclusion criteria, exposure and outcome measurement, confounder handling, and statistical analysis. For studies comparing interventions (e.g., screening methods), the ROBINS-I risk tool will ascertain the risk of bias across seven domains: selection, confounding, intervention classification, missing data, deviations, outcome measurement, and selective reporting [23,24]. Two review authors will independently apply the JBI appraisal tool and ROBINS-I to assess the internal validity of included studies. The JBI tool, designed for cross-sectional studies, evaluates eight criteria: clear inclusion criteria, detailed study description, valid exposure measurement, standard outcome criteria, confounder identification, confounder handling, valid outcome measurement, and appropriate statistical analysis. Responses will be rated as Yes, No, Unclear, or Not Applicable, with narrative synthesis determining overall risk of bias. ROBINS-I, developed for non-randomized studies of interventions in systematic reviews [23,24], rates bias as Low, Moderate, Serious, or Critical per domain. Studies with multiple No/Unclear JBI responses or Serious/Critical ROBINS-I ratings will be considered high risk of bias, informing GRADE assessments.

Inconsistency will be assessed by examining variability in outcome estimates across studies, with substantial heterogeneity (e.g., due to differences in screening methods or populations) prompting downgrading unless explained by planned subgroup analyses (e.g., by screening method, programme type, or geographical setting). Indirectness will be evaluated to ensure evidence aligns with the review’s population (Ghanaian women), interventions (e.g., VIA, HPV DNA testing, cryotherapy), and outcomes. Imprecision will be assessed based on sample sizes and the availability of confidence intervals, with small or variable samples leading to downgrading. Publication bias will be explored narratively, considering the potential for selective reporting in grey literature, and, where meta-analysis is feasible, using Egger’s test for continuous outcomes or Begg’s test for binary outcomes.

Discrepancies between review authors regarding the risk of bias assessments for individual studies will be investigated through structured discussion until a consensus is found. If consensus cannot be achieved, a third independent review author will be invited to adjudicate and provide a final decision. Results will be presented in a GRADE Summary of Findings table, detailing the certainty of evidence for each outcome (High, Moderate, Low, Very Low), and reported following PRISMA guidelines to ensure transparency.

### Data extraction, management and ethics

Ethical approval was not required for the present study. Data from included full-text articles will be systematically extracted using a standardized, pre-piloted data extraction form designed to capture all relevant study characteristics and outcomes. Bibliographic details, including manuscript title, author names, publication date, and Digital Object Identifier (DOI), will be recorded to ensure accurate citation and traceability. Key study characteristics, such as sample size, target population, data collection period, geographical location, study objectives, eligibility criteria, and methodological design elements, will also be extracted to contextualize the research. Additionally, specific data related to cervical precancer screening and treatment approaches will be collected, including screening modalities, treatment interventions, and associated outcomes (e.g., screening uptake, diagnostic accuracy, treatment efficacy, or adverse events). All extracted data will be entered into a structured format and stored securely on an institutional server with regular offline backups to ensure data integrity and compliance with data protection standards.

### Presentation of data and results

An accounting of the search procedure and report screening approach will be presented in the updated PRISMA flow chart [19]. Data and information obtained from a review of available literature will be put together and represented with tables, maps and figures for ease of comparison. Aggregated data from selected studies will be summarized using descriptive statistics.

### Statistical analysis

Given the anticipated diversity in study designs (cross-sectional studies, baseline surveys of prospective studies, and programme reports), screening methods (e.g., visual inspection with acetic acid [VIA], HPV DNA testing, mobile colposcopy, cytology), and outcome measures (e.g., screening coverage, VIA positivity rates, high-risk HPV [hr-HPV] prevalence, treatment outcomes, loss to follow-up rates), we expect limited scope for meta-analysis. However, where studies report comparable outcomes (e.g., hr-HPV prevalence or VIA positivity rates) using similar screening methods and populations, we will explore the feasibility of pooling results using meta-analytic techniques. For studies with sufficiently homogeneous data, we will use R studio software to conduct a random-effects meta-analysis, as this approach accounts for expected between-study variability in effect sizes. For binary outcomes (e.g., proportion of women treated, loss to follow-up rates), we will compute risk ratios (RRs) or odds ratios (ORs) with 95% CIs, depending on the data reported. Two-sided p-values will be calculated for each outcome to assess statistical significance. To account for potential clustering effects in studies conducted across multiple regions or screening centres, we will adjust standard deviations for the design effect if intra-class correlation coefficients (ICCs) are provided in the study reports. If ICCs are not reported, we will use external estimates from similar cervical cancer screening and prevention studies in low-resource settings. Heterogeneity between studies will be assessed using the chi-square test for heterogeneity and the I^2^ statistic. An I^2^ value greater than 50% will be considered indicative of substantial heterogeneity, prompting further investigation. To explore sources of heterogeneity, we will conduct sensitivity analyses based on study quality, as assessed by the ROBINS-I and JBI risk appraisal tools. Studies with high risk of bias will be compared to those with low to moderate risk to evaluate the robustness of findings. We will perform subgroup analyses to investigate heterogeneity in effect estimates according to study quality, screening method, programme type, geographical setting and population characteristics.

For outcomes where meta-analysis is not feasible due to heterogeneity or insufficient data, we will conduct a descriptive synthesis. This will involve summarizing key findings in tables, figures, and narrative text, focusing on screening coverage, positivity rates, hr-HPV prevalence, treatment outcomes, and loss to follow-up rates. Programme performance will be evaluated using the RE-AIM framework (Reach, Effectiveness, Adoption, Implementation, Maintenance), with scores compared across programmes to identify contextual strategies with the greatest impact.

To assess potential publication bias, we will use Egger’s weighted regression test for continuous outcomes (e.g., hr-HPV prevalence) and Begg’s rank correlation test for dichotomous outcomes (e.g., direct visual inspection outcomes), provided at least 10 studies report the same outcome. Funnel plots will be visually inspected for asymmetry if meta-analysis is performed. For grey literature such as programme reports, we will narratively assess the risk of selective reporting by using the AACODS checklist as indicated above.

### Study timeline and status

This review started in March 2025 with protocol design, search strategy optimization, and registration in PROSPERO (CRD420251035338). We expect to complete it and report results in 9–12 months: see Supporting information file 2 (S2 Table) for detailed timeline. Record screening, including title/abstract and full-text screening, is expected to commence in August 2025 in line with peer-reviewer feedback on study protocol. Data extraction using a standardized form is expected to be completed by October 2025, based on the number of included studies and complexity of data. The final synthesis, meta-analysis, and reporting of results are expected by January 2026. Any timeline updates will be documented in the PROSPERO entry.

## Conclusion

This review will systematically update our understanding of the impact and performance of cervical screening programmes in Ghana. The systematic approach will produce a comprehensive synthesis of evidence from available published reports and identify gaps that require urgent attention for cervical cancer elimination. Results will be presented in accordance with the Preferred Reporting Items for Systematic Reviews and Meta-Analyses (PRISMA) guidelines to ensure transparency and reproducibility. Finally, we will specifically explore programmatic strategies and approaches used and highlight which approaches are likely to yield the most impact in similar settings.

## Supporting information

S1 Table PRISMA-P (Preferred Reporting Items for Systematic Review and Meta-Analysis Protocols) 2015 checklist: recommended items to address in a systematic review protocol. (DOCX)

S2 Table Timeline and status of systematic review (CRD420251035338). (DOCX)

## Abbreviations

CCPTC: Cervical Cancer Prevention and Training Centre
CECAP: Climate and Ecological Crisis Action Plan
CINAHL: Cumulated Index in Nursing and Allied Health Literature
DNA: Deoxyribonucleic acid
EVA: Enhanced visual assessment
GHS: Ghana Health Service
HPV: Human papilloma virus
hr-HPV: High risk human papilloma virus
JHPIEGO: Johns Hopkins Programme for International Education in Gynaecology and Obstetrics
LEEP: Loop electrosurgical excision procedure
MeSH: Medical subject headings
NIH: National Institutes of Health
PLHIV: People living with Human immunodeficiency virus
PRISMA: Preferred Reporting Items for Systematic Reviews and Meta-Analyses
RE-AIM: Reach, effectiveness, adoption, implementation, and maintenance
SAFE: Safety, Accessibility, Feasibility and Programme Effort
SVA: Single visit approach
USAID: United States Agency for International Development
VIA: Visual inspection with acetic acid
WHO: World Health Organization
