# Supplementary material for: Protocol for a systematic review and meta-analysis of the impact of cervical cancer prevention initiatives in Ghana
Source: PLoS One. 2025 Nov 20;20(11):e0337011. doi: 10.1371/journal.pone.0337011 (PMC12633947; doi:10.1371/journal.pone.0337011)
Supplement: S2 Table — (DOCX) [file pone.0337011.s002.docx]

| **Activity/Task** | **Start Date** | **End Date** | **Duration** | **Status** |
| --- | --- | --- | --- | --- |
| Protocol Design | March 1, 2025 | March 31, 2025 | 1 month | Completed |
| Search Strategy Optimization | March 1, 2025 | March 31, 2025 | 1 month | Completed |
| PROSPERO Registration (CRD420251035338) | March 1, 2025 | March 31, 2025 | 1 month | Completed |
| Literature Search Execution | May 1, 2025 | July 31, 2025 | 3 months | Optimizing |
| Record Screening (Title/Abstract & Full-Text) | August 1, 2025 | October 31, 2025 | 3 months | Planned |
| Data Extraction | October 1, 2025 | October 31, 2025 | 1 month | Planned |
| Quality Assessment/Risk of Bias | November 1, 2025 | November 30, 2025 | 1 month | Planned |
| Stakeholder Consultation | December 1, 2025 | December 31, 2025 | 1 month | Planned |
| Final Synthesis, Meta-Analysis, and Results Reporting | November 1, 2025 | January 31, 2026 | 3 months | Planned |

**S2 Table: Timeline and Status of Systematic Review (CRD420251035338)**
